# Xinjiang Brown Cattle-Derived β-Defensin EBD Alleviates *Enterococcus faecalis*-Induced Mammary Inflammation in Mice

**DOI:** 10.3390/cells15211980

**Published:** 2026-10-30

**Authors:** Yaolong Song, Yingchao Li, Mingshuai Chen, Chenyang Shi, Qirui Zang, Wanjing Jin, Qian Ma, Binhuan Ma, Panpan Tong, Shicheng Wan, Aili Aierken, Mengfei Zhang

**Affiliations:** 1 College of Veterinary Medicine, Xinjiang Agricultural University, Urumqi 830052, China; 17726785228@163.com (Y.S.); liyingchao0409@163.com (Y.L.); chenms416@outlook.com (M.C.); 17634384908@163.com (C.S.); 19199130252@163.com (Q.Z.); 13319912981@163.com (W.J.); 15339306406@163.com (Q.M.); 15750679553@163.com (B.M.); tongpanpan@xjau.edu.cn (P.T.); 2 Urumqi Field Scientific Observation and Research Station for Animal Diseases, Ministry of Agriculture and Rural Affairs, Urumqi 830052, China; 3 Xinjiang Key Laboratory of New Drug Research and Development for Herbivorous Animals, Urumqi 830052, China; 4 Xinjiang Regional Key Laboratory of Clinical Veterinary Medicine Research, Urumqi 830052, China; 5 Herbivorous Animal Biomedicine and Diagnostic Technology Engineering Research Center, Xinjiang Uygur Autonomous Region, Urumqi 830052, China; 6 College of Veterinary Medicine, Northwest A&F University, Yangling 712100, China; shicheng_wan@nwafu.edu.cn; 7 State Key Laboratory of Pathogenesis, Prevention and Treatment of High Incidence Diseases in Central Asia, Xinjiang Medical University, Urumqi 830011, China; alijan@xjmu.edu.cn

**Keywords:** Xinjiang Brown Cattle, bovine enteric β-defensin, *Enterococcus faecalis*, bovine mastitis, antibacterial effect, eukaryotic expression, gene regulation

## Abstract

Bovine mastitis is a major infectious disease in dairy cattle, and the increasing prevalence of antimicrobial-resistant pathogens has created an urgent need for the development of effective antimicrobial agents. In this study, bovine enteric β-defensin (EBD) from Xinjiang Brown Cattle was cloned, expressed, and preliminarily evaluated for its antibacterial and protective effects against *Enterococcus faecalis*-induced mastitis. Bioinformatic analysis identified EBD as a secreted protein belonging to the bovine β-defensin family. The recombinant PCDH-EBD construct was successfully transfected into 293T cells, resulting in markedly increased EBD mRNA expression and detectable recombinant EBD protein expression, as confirmed by SDS-PAGE and Western blotting. In vitro agar diffusion assays showed that the EBD-containing preparation exhibited inhibitory activity against *E. faecalis*. In a murine mastitis model, EBD treatment significantly attenuated mammary histopathological lesions and reduced the expression of *Mpo*, *Tnf*, and *Il6* compared with the mastitis group. 16S rRNA sequencing of mammary gland tissues showed that EBD treatment was associated with a reduced relative abundance of *Enterococcus* and partial restoration of microbial diversity. Transcriptomic analysis further revealed that EBD treatment was associated with alterations in NOD-like receptor signaling- and phagosome-related pathways, suggesting that these processes may contribute to its protective effects. Collectively, these findings indicate that enteric β-defensin EBD from Xinjiang Brown Cattle possesses antibacterial and anti-inflammatory potential, making it a candidate for developing antimicrobial agents against bovine mastitis.

## 1. Introduction

Bovine mastitis is one of the most prevalent and economically important infectious diseases affecting the dairy industry worldwide, resulting in substantial losses in milk yield and quality, increased veterinary costs, premature culling, and reduced farm profitability [1]. Clinically, mastitis is characterized by swelling, redness, pain, and impaired secretory function of the mammary gland, often accompanied by abnormal milk, including clots, flakes, or watery secretions. In severe cases, systemic manifestations such as fever, anorexia, and depression may also occur [2]. The disease is caused by a wide range of contagious and environmental pathogens, among which *Staphylococcus aureus*, *Streptococcus* spp., *Escherichia coli*, and *Enterococcus faecalis* (*E. faecalis*) are considered major bacterial etiological agents [3]. The increasing prevalence of antimicrobial-resistant mastitis pathogens has substantially reduced the efficacy of conventional antibiotic therapy, while the extensive use of antimicrobials in dairy production has also raised concerns regarding antimicrobial residues in milk and the associated risks to food safety and public health [4]. Consequently, the development of safe and effective alternatives to conventional antibiotics has become an important research priority for the prevention and control of bovine mastitis.

*Enterococcus faecalis* is an opportunistic pathogen associated with bovine mastitis [5]. Its virulence-associated factors, biofilm-forming ability, and antimicrobial resistance can complicate infection control [6,7]. Therefore, investigating host-derived antimicrobial peptides as potential protective agents against *E. faecalis*-associated mammary inflammation is of considerable interest. Under adverse environmental or physiological stress conditions in cattle, *E. faecalis* expresses multiple virulence determinants, including lipoteichoic acid (LTA), peptidoglycan, aggregation substance, surface adhesins, sex pheromones, and hydrolytic enzymes, thus triggering inflammatory responses and promoting tissue damage [7]. It is also involved in biofilm formation and regulates transcriptional networks associated with virulence and stress adaptation genes, ultimately contributing to the development of bovine mastitis. In recent years, the incidence of mastitis caused by drug-resistant *E. faecalis* strains arising from antibiotic misuse has increased on a year-to-year basis [3].

β-Defensins are small cationic antimicrobial peptides that constitute an important component of the innate immune system in mammals. In addition to their direct antimicrobial activity against bacteria, fungi, and certain viruses, β-defensins have been reported to modulate inflammatory responses, recruit immune cells, and contribute to the maintenance of epithelial barrier integrity, thereby playing important roles in host defense and immune homeostasis [8,9]. Among bovine β-defensins, enteric β-defensin (EBD) has attracted increasing attention because of its potential antimicrobial and immunomodulatory activities. However, its biological function, particularly its protective role against *Enterococcus faecalis*-associated bovine mastitis, remains poorly understood [10]. Xinjiang Brown Cattle are a locally developed breed adapted to the environmental and husbandry conditions of Xinjiang. The oral mucosal surface, serving as the primary barrier against pathogen entry, relies on β-defensins and related antimicrobial peptides (AMPs) to maintain an effective immune defense [11,12]. EBD, a member of the bovine β-defensin family [13], is closely associated with mucosal immune function in Brown Cattle, particularly regarding its gene sequence characteristics, protein structural and functional attributes, and in vivo expression profiles [14]. However, the antibacterial activity of Xinjiang Brown Cattle-derived EBD against *E. faecalis* and its potential protective effects on mammary inflammation remain insufficiently characterized [15].

In this study, we cloned and expressed Xinjiang Brown Cattle-derived EBD and evaluated its preliminary antibacterial activity in vitro. We further investigated its potential protective effects against *E. faecalis*-induced mammary inflammation in a murine model through histopathological, inflammatory gene expression, microbiome, and transcriptomic analyses.

## 2. Materials and Methods

### 2.1. Cloning of Bovine β-Defensin EBD

Fresh bovine tissue was obtained from a Brown Cattle abattoir in Changji, Xinjiang. Samples were immediately transferred into enzyme-free, sterile tubes containing 1 mL of TRIzol reagent and then immersed in liquid nitrogen to prevent RNA degradation. On ice, tissues were thoroughly homogenized in the same tubes with a pestle; 200 μL of chloroform was added, and the mixture was vortexed on a room-temperature shaker, then incubated on ice for 10 min. Samples were then centrifuged at 12,000 r/min for 10 min at 4 °C. The upper aqueous phase was carefully transferred to a new enzyme-free sterile tube, mixed with an equal volume of isopropanol, vortexed for 15 s, and incubated on ice for 50 min. After centrifugation at 12,000 r/min for 15 min at 4 °C, the supernatant was removed. The RNA pellet was washed with 1 mL absolute ethanol, centrifuged for 3 min, and the supernatant discarded; this washing step was repeated three times. Residual ethanol was air-dried in a laminar flow cabinet, and the RNA was dissolved in 50 μL RNase-free water. RNA concentration was determined by spectrophotometry.

First-strand cDNA was synthesized using PrimeScript™ IV 1st Strand cDNA Synthesis Mix (Takara Bio Inc., Kusatsu, Shiga, Japan; Cat. No. 6215A). The reaction system contained 4 μL 5 × PrimeScript IV cDNA Synthesis Mix, 2 μL Random 6-mers, 3 μL RNA template (5 μg), and 11 μL RNase-free ddH_2_O. The reverse-transcription conditions were as follows: 30 °C for 10 min, 42 °C for 15 min, and 70 °C for 15 min.

Using cDNA as a template and EBD-specific cloning primers (EBD-K-F: 5′-ATGAGGCTCCATCACCTGCTCCT-3′; EBD-K-R: 5′-TTACCTCCACCTGCAGCATT TTACTG-3′), PCR amplification was performed using PrimeSTAR Max high fidelity DNA polymerase (Takara Bio Inc., Kusatsu, Shiga, Japan; Cat. No. R045A). PCR products were separated on a 2% agarose gel; target bands were excised and purified using a Tiangen gel extraction kit (TIANGEN Biotech (Beijing) Co., Ltd., Beijing, China; Cat. No. DP209), then stored at −20 °C until further use.

### 2.2. Construction of the Eukaryotic Expression Vector and Plasmid Identification

The eukaryotic expression vector PCDH was linearized by double digestion with XbaI (Cat. No. R0145, New England Biolabs, Ipswich, MA, USA) and BamHI (Cat. No. R0136; New England Biolabs, Ipswich, MA, USA), verified on a 1% agarose gel, and the linearized fragment was recovered using a Tiangen gel extraction kit. The gel-purified product from Section 2.1 was used as a PCR template with homology arm primers (TY-EBD-F: 5′-ACCTCCATAG AAGATTCTAGAATGAGGCTCCATCACCTGCTCCT-3′; TY-EBD-R: 5′-ATCCTTCG CGGCCGCGGATCCTTACCTCCACCTGCAGCATTTTACTG-3′) to introduce flanking homology sequences. PCR products were separated on a 2% agarose gel; target bands were excised and purified. The purified insert fragment and linearized PCDH vector were ligated using a homologous recombination cloning kit.

Ten microliters of recombinant plasmid PCDH-EBD were added to 100 μL of *E. coli DH5α* competent cells and incubated on ice for 30 min, followed by heat shock at 42 °C for 90 s and immediate incubation on ice for 2 min. Then, 900 μL of antibiotic-free LB liquid medium was added, and the cells were incubated at 37 °C and 180 r/min for 50 min. After centrifugation at 5000 r/min for 5 min, 900 μL of supernatant was removed, and the remaining cell suspension was spread onto LB agar plates containing 100 μg/mL ampicillin and incubated overnight at 37 °C. Ten colonies were picked and cultured in 25 mL of LB broth supplemented with 100 μg/mL ampicillin at 37 °C and 180 r/min for 6 h. Colony PCR was performed, and products were analyzed on 1% agarose gels to identify positive clones. Plasmid DNA from confirmed clones was extracted using a Tiangen miniprep kit, subjected to Sanger sequencing, and verified against the EBD reference sequence using DNAMAN software (version 6.0.3.99).

### 2.3. Bioinformatic Analysis of Bovine β-Defensin EBD

Online tools, including ProtParam 1.0, TMHMM 2.0, and SignalP 5.0, were used for systematic bioinformatics analysis of bovine β-defensin EBD, covering its physicochemical properties, hydrophilicity/hydrophobicity profile, transmembrane domain prediction, and signal peptide characteristics.

### 2.4. Cell Transfection

The 293T human embryonic kidney cells used in this study were purchased from BeNa Culture Collection (BNCC), China, with a catalog number of BNCC353535. The recombinant PCDH-EBD plasmid was transfected into 293T cells using Lipo6000 reagent. Three independent transfections were performed. Thirty minutes prior to transfection, the 5% serum-supplemented DMEM was replaced with serum-free DMEM for serum starvation. Two enzyme-free, sterile 1.5 mL centrifuge tubes were prepared, each containing 125 μL serum-free DMEM. One tube was supplemented with 5 μL Lipo6000 transfection reagent, and the other with 2.5 μg recombinant PCDH-EBD plasmid, followed by separate incubation at room temperature for 20 min. The plasmid solution was then transferred into the tube containing transfection reagent, mixed gently, and incubated for an additional 5 min. Ten minutes before transfection, the cell culture medium was replaced with 5% serum-containing complete medium. The transfection complex was added dropwise to the culture dish, and parallel transfection with empty PCDH vector was set as the control. After incubation at 37 °C under 5% CO_2_ for 6 h, the medium was refreshed with complete medium. At 24 h post-transfection, successfully transfected cells were screened with 5 μg/mL puromycin for 24 h. The selection medium was discarded, and fresh complete medium was replenished for another 48 h of culture before cell morphology and density assessment. Adherent cells were digested with 0.25% trypsin for 3 min, resuspended, collected into sterile 15 mL centrifuge tubes, and centrifuged at 1500 r/min for 3 min. The supernatant was discarded, and the cell pellet was washed with 1 mL PBS, followed by another centrifugation at 1500 r/min for 3 min. One aliquot of cell pellet was stored at −80 °C for protein analysis; the other aliquot was treated with 1 mL TRIzol reagent to extract total RNA, which was subsequently reverse-transcribed into cDNA and stored at −20 °C for subsequent use.

### 2.5. Quantitative Real-Time PCR Detection

For evaluation of recombinant EBD expression, total RNA was extracted from HEK293T cells transfected with PCDH-EBD or the corresponding control vector. For analysis of the murine mastitis model, total RNA was isolated from mammary gland tissues collected from mice in the NC, Mastitis, and EBD-treated groups. The expression levels of *Mpo*, *Tnf*, *Il6*, *Il1b*, *Cxcl1*, *Tjp1*, *Ocln*, and *Bcl2* were determined by RT-qPCR. The empty-vector PCDH group served as the negative control, and *Actb* was used as the reference gene for normalization. EBD-specific primers were used to assess recombinant plasmid-derived EBD transcript expression in transfected cells. The primer sequences used for qPCR analysis are listed in Table 1.

Each 20 μL reaction contained SYBR Green qPCR mix, specific primers, reverse-transcribed cDNA, and ddH_2_O to the final volume. The amplification program consisted of an initial denaturation at 95 °C for 30 s, followed by 40 cycles of denaturation at 95 °C for 5 s and annealing at 60 °C for 30 s. Following amplification, melting curve analysis was performed to confirm the specificity of the amplification products. Relative gene expression levels were calculated using the 2^−ΔΔCt^ method.

### 2.6. Total Cellular Protein Extraction

Protein samples were prepared from HEK293T cells transfected with PCDH-EBD and the corresponding empty vector. Frozen cell pellets were thawed on ice and lysed with 300 μL RIPA buffer containing 2% protease inhibitor, 2% phosphatase inhibitor, and 2% 0.05 M EDTA. Samples were incubated on ice for 30 min with pipetting every 5 min. Lysates were centrifuged at 12,000 r/min for 10 min at 4 °C, and the supernatants were transferred to fresh enzyme-free tubes. The recombinant protein preparation was purified using the HisPur™ Ni-NTA Spin Purification Kit (Thermo Fisher Scientific, Waltham, MA, USA; Cat. No. 88229), and protein concentration was determined using a BCA Protein Assay Kit (Cat. No. P0012; Beyotime Biotechnology, Shanghai, China). The resulting EBD-containing preparation was used in subsequent in vitro and in vivo experiments. Then, 5× SDS loading buffer was added, mixed by pipetting, heated at 100 °C for 10 min in a metal bath, cooled to room temperature, and stored at −20 °C. The denatured protein was subjected to Western blot analysis and detection with an anti-His-tag antibody.

### 2.7. SDS-PAGE and Western Blot Analysis

Protein gels were prepared using a rapid SDS-PAGE gel preparation kit (Cat. No. P0012AC; Beyotime Biotechnology, Shanghai, China), and 20 μL of each protein sample and 5 μL of molecular weight marker were loaded per lane. Following electrophoresis, the stacking and resolving gels were separated. For Coomassie Brilliant Blue staining, the resolving gel was immersed in deionized water and microwaved to boiling for 60 s, after which the water was discarded. Subsequently, 75 mL of rapid Coomassie Brilliant Blue staining solution was added, and the gel was microwaved to boiling for another 60 s. The staining solution was then removed and replaced with 75 mL of fresh deionized water, followed by an additional 60 s of microwave treatment. The gel was destained overnight in deionized water on a horizontal shaker at 50 rpm and subsequently washed with deionized water until the background was sufficiently clear. Band intensities were quantified using ImageJ software v1.43 to evaluate EBD protein expression.

For Western blotting, following SDS-PAGE, proteins were transferred onto a methanol-activated PVDF membrane at 90 V for 60–90 min. The membrane was blocked with 5% skim milk for 2 h and subsequently incubated overnight at 4 °C with anti-His-tag (AH367, Beyotime Biotechnology, Shanghai, China; 1:1000). After washing with PBST, the membrane was incubated with HRP-conjugated goat anti-mouse IgG (A0216, Beyotime; 1:5000) for 1 h at room temperature. Following further washing, protein bands were visualized using a chemiluminescent substrate and imaged using a chemiluminescence imaging system.

### 2.8. In Vitro Antibacterial Assay of Recombinant EBD Protein

Clinical *Enterococcus faecalis* isolates obtained from bovine mastitis cases were used together with five reference bacterial strains, including *Escherichia coli* ATCC 25922, *Salmonella enterica* subsp. *enterica* serovar Typhimurium ATCC 14028, *Staphylococcus aureus* ATCC 29213, methicillin-resistant *Staphylococcus aureus* (MRSA) ATCC 43300, and *Salmonella enterica* subsp. *enterica* serovar *Enteritidis* ATCC 13076. The clinical *Enterococcus faecalis* isolate was identified via 16S rRNA sequencing and whole-genome sequencing. The five reference strains were purchased from Xinjiang Yigen Biology Science and Technology Co., Ltd. (Urumqi, China). *Enterococcus faecalis* is a widely prevalent opportunistic pathogen recognized to cause clinical and subclinical mastitis in dairy cows, severely compromising cow health and farming profitability.

The antibacterial activity of EBD protein was evaluated using the agar well diffusion method. Briefly, 100 µL of each standardized bacterial suspension was evenly spread onto Mueller-Hinton agar plates. Wells were subsequently prepared in the agar, and 50 μL of ddH_2_O (blank control), 50 µL of EBD protein (1 mg/mL), 50 µL of PCDH protein (1 mg/mL, negative control), or 50 µL of kanamycin solution (1 mg/mL; positive control). The EBD concentration (1 mg/mL) was selected based on previous studies of β-defensins and cationic antimicrobial peptides, as well as preliminary range-finding experiments in our laboratory. At this concentration, EBD produced reproducible inhibition zones without visible precipitation or nonspecific effects. The plates were incubated at 37 °C for 12 h, after which the diameters of the inhibition zones were measured using a vernier caliper. The 12 h incubation period was used for preliminary evaluation of antibacterial activity rather than Clinical and Laboratory Standards Institute (CLSI)-standardized antimicrobial susceptibility testing, thereby minimizing excessive bacterial overgrowth. The agar well diffusion assay was performed in five independent experiments for each bacterial strain, with one measurement per strain in each experiment. Inhibition-zone diameters are presented as mean ± SD (*n* = 5). Values are presented as mean ± SD. Antibacterial activity was classified according to the inhibition zone diameter: <8 mm, negative (−); 8–12 mm, weak (+); 12–16 mm, moderate (++); and >16 mm, strong (+++).

### 2.9. Determination of E. faecalis Colony-Forming Units (CFU)

*E. faecalis* was cultured in a shaking incubator at 37 °C until the OD_600_ reached 0.6. The bacterial culture was serially diluted to 10^−3^, 10^−4^, and 10^−5^, and each dilution was plated in triplicate onto LB agar plates. Three independent bacterial cultures were used. The plates were incubated at 37 °C overnight. Following incubation, colonies were enumerated, and plates containing 30–300 colonies were selected for CFU determination. The bacterial concentration was calculated using the following formula: CFU/mL = Mean colony count × Dilution factor.

### 2.10. Establishment of the Murine Mastitis Model and Treatment Protocol

Fifty 8-week-old female and twenty-five 8-week-old male Kunming (KM) mice (20–25 g) were purchased from Servicebio (Urumqi, China). All animals were housed in SPF-grade facilities under controlled conditions (22 ± 2 °C, 50–60% relative humidity, and a 12 h light/dark cycle). Housing arrangements varied by reproductive stage: one male was paired with two to three females per cage during mating; after pregnancy confirmation, males were removed and pregnant females were housed individually or in small same-sex groups; following parturition, lactating females were housed individually with their litters. All cages contained autoclaved bedding, and mice had free access to standard chow and sterile water. After 7 days of acclimatization, natural mating was performed.

Of the 50 female mice initially enrolled in the breeding program, 15 lactating mice that met the predefined inclusion criteria were selected for the mastitis experiment. The remaining female mice were excluded owing to non-pregnancy or delayed parturition. The 15 selected mice were randomly assigned to three groups: NC group, mastitis group and EBD treatment group, with 5 mice per group. The murine mastitis model was established by intramammary inoculation with minor modifications to a previously described protocol (Ji et al., 2023) [16]. Both the left (L4) and right (R4) fourth inguinal mammary glands of each mouse received the same treatment. Each gland received a total volume of 100 μL. In the negative-control group, each gland received 100 µL sterile physiological saline. In the mastitis group, each gland received 50 µL of an *Enterococcus faecalis* suspension (6.7 × 10^6^ CFU/mL) plus 50 µL sterile physiological saline. In the EBD-treated group, each gland received 50 µL of the same *E. faecalis* suspension plus 50 µL EBD protein (1 µg/µL). Thus, each infected mammary gland received 3.35 × 10^5^ CFU of *E. faecalis*, corresponding to a total inoculum of 6.7 × 10^5^ CFU per mouse. The final bacterial concentration in each 100 μL injection mixture was 3.35 × 10^6^ CFU/mL. Each EBD-treated gland received 50 µg EBD protein. Body weight was monitored during the breeding and peripartum periods. Following intramammary bacterial challenge, clinical observations were conducted at 12 h intervals over a 24 h period. At 24 h post-injection, mice were euthanized by gradual-fill carbon dioxide inhalation followed by cervical dislocation to ensure death, and both injected mammary glands were collected. For histopathology, each gland was processed separately; five non-overlapping fields per gland were scored, and the mean score of the two glands was used as the value for each mouse. For qRT-PCR, 16S rRNA sequencing, and RNA-seq, equal amounts of tissue from the two injected glands were combined as one sample per mouse. Histopathological lesions of mammary tissues was evaluated based on four indicators: inflammatory cell infiltration, acinar structure destruction, interstitial edema, and tissue necrosis. Each indicator was scored from 0 to 3: 0, no lesion; 1, mild focal changes; 2, moderate multifocal lesions; 3, severe diffuse lesions. For each mouse, five non-overlapping fields of view at 200-fold magnification were randomly selected within standardized mammary tissue regions. The total pathological score per mouse (range, 0–12) was calculated by summing the scores of the four indicators. All sections were coded and scored in a blinded manner by an investigator unaware of group assignments.

### 2.11. Microbiome Bioinformatics Analysis of Mastitis

Following euthanasia, mammary gland tissues were aseptically collected from all five animals in each group in a biosafety cabinet to minimize environmental contamination. Samples were shipped to Novogene on dry ice for DNA extraction, library construction, and sequencing. A prespecified subset of four mice per group (*n* = 4 per group; 12 total) was used for 16S rRNA sequencing. These mice were selected from the 15 randomized dams and were not excluded from the primary animal experiment. and the selection was performed without reference to histopathological, qRT-PCR, or sequencing outcomes.

Total microbial DNA was extracted, and the V3–V4 region of the bacterial 16S rRNA gene was amplified using the primers 5′-CCTAYGGGRBGCASCAG-3′ and 5′-GGACTACNNGGGTATCTAAT-3′. Libraries were sequenced on an Illumina NovaSeq platform (Illumina, Inc., San Diego, CA, USA) using a PE250 strategy. After barcode and primer removal using cutadapt (v3.3), paired-end reads were merged with FLASH (v1.2.11), quality-filtered using fastp (v0.23.1), and screened for chimeras using UCHIME in vsearch (v2.16.0). ASVs were inferred using DADA2 in QIIME2 (v2022.02), and taxonomic annotation was performed using the SILVA 138.1 database.

For diversity and taxonomic composition analyses, ASV abundance was normalized to the sequencing depth of the sample with the fewest sequences. Differential abundance analysis was performed separately on unrarefied count data using ANCOM-BC2 implemented in the ANCOMBC R package (version 2.14.0) in R (version 4.6.1). ASV counts were aggregated at the genus level, and taxa lacking genus-level annotation or assigned to chloroplasts or mitochondria were excluded. Genera detected in fewer than 25% of samples were removed. Global and pairwise comparisons among the WT, Mastitis, and EBD-treated groups were performed with WT as the reference group. *p* values were adjusted using the Benjamini–Hochberg FDR procedure, and pseudo-count sensitivity analysis was used to assess robustness. Genera with q < 0.05 that passed the sensitivity analysis were considered robust differentially abundant taxa.

### 2.12. RNA-Seq and Data Analysis

Total RNA was extracted from mammary gland tissues using TRIzol reagent. A prespecified subset of three mice per group (*n* = 3 per group; 9 total) was used for RNA-seq. These mice were selected from the 15 randomized dams, with each mouse representing one independent biological replicate. RNA integrity was evaluated using an Agilent 2100 Bioanalyzer (Agilent Technologies, Santa Clara, CA, USA), and only RNA samples with an RNA integrity number (RIN) ≥ 7.0 were used for library construction. 3000 ng of total RNA per sample was used as input for library preparation. RNA-sequencing libraries were prepared by Novogene (Beijing, China) according to the service provider’s standard protocol. Library quality was assessed using an Agilent 2100 Bioanalyzer. After library quality assessment, the libraries were pooled and sequenced on an Illumina NovaSeq platform to generate 150 bp paired-end reads.

All sequencing raw reads were processed with strict quality control to eliminate adapter sequences, ambiguous base-containing reads, and low-quality reads. Qualified clean reads were subsequently mapped to the mouse reference genome Ensembl_100_mus_musculus_grcm38_toplevel using HISAT2 software (version 2.0.5), and the corresponding genome and gene annotation files were retrieved from Ensembl release 100.

Gene-level read counts were generated using featureCounts (v1.5.0-p3), and differential-expression analysis was performed using DESeq2 (v1.20.0). Genes with |log2FoldChange| ≥ 1 and Benjamini–Hochberg-adjusted *p*-values ≤ 0.05 were considered differentially expressed. Identified differentially expressed genes were further subjected to Gene Ontology (GO) functional enrichment analysis and Kyoto Encyclopedia of Genes and Genomes (KEGG) pathway enrichment analysis to identify biological functions and signaling pathways potentially associated with the effects of EBD in *E. faecalis*-induced mastitis. All libraries were prepared and sequenced in a single batch, and no batch correction was therefore applied. No samples that met the predefined RNA-quality criteria were excluded from downstream RNA-seq analysis.

### 2.13. Statistical Analysis

Statistical analyses of experimental data were performed using GraphPad Prism v8.0.2 and ImageJ software v1.43. Comparisons between two independent groups were performed using a two-tailed unpaired Student’s *t*-test. For comparisons involving three groups, one-way analysis of variance (ANOVA) followed by Tukey’s multiple-comparison test was performed when the data satisfied the assumptions of normality and homogeneity of variance. When these assumptions were not met, the non-parametric Kruskal–Wallis test followed by Dunn’s multiple-comparison test was used. Statistical significance was defined as *p* < 0.05. For RNA-seq analysis, differentially expressed genes (DEGs) were defined using |log_2_FoldChange| ≥ 1 and a Benjamini–Hochberg-adjusted *p* value ≤ 0.05. The individual mouse was considered the experimental unit for animal-based analyses. No randomized dams were excluded from the primary animal experiment, histopathological analysis, or qRT-PCR analysis. Prespecified subsets were used for 16S rRNA sequencing (*n* = 4 per group) and RNA-seq (*n* = 3 per group).

## 3. Results

### 3.1. Cloning and Bioinformatic Analysis of Bovine β-Defensin EBD

**Figure 1:**
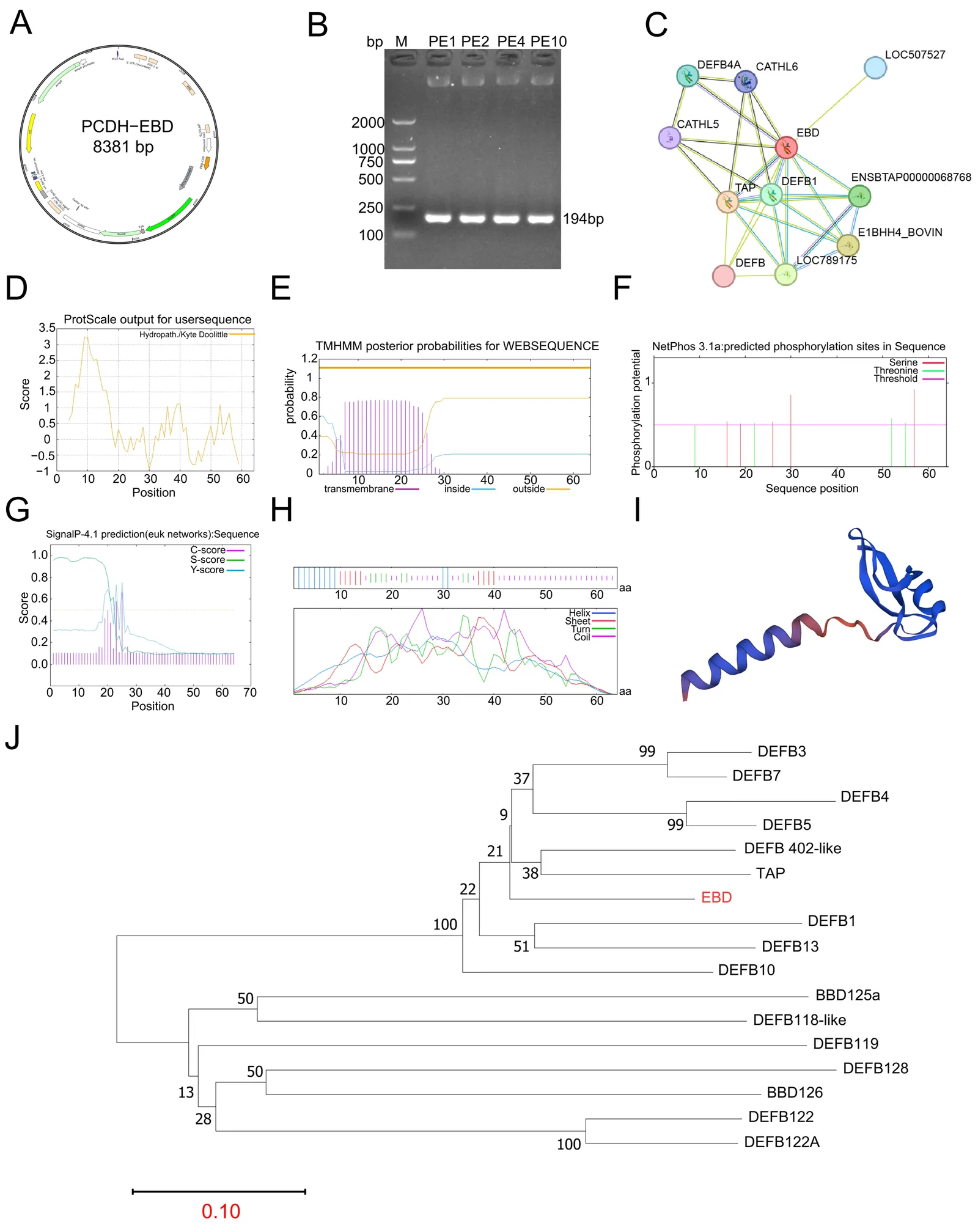
Identification and bioinformatic analysis of recombinant PCDH-EBD. (**A**) Schematic representation of the recombinant PCDH-EBD plasmid map; (**B**) PCR-based identification of recombinant plasmids; (**C**) EBD genomic context and neighboring gene analysis; (**D**) Hydrophilicity/hydrophobicity profile of EBD; (**E**) Prediction of transmembrane domains; (**F**) Prediction of phosphorylation sites; (**G**) Signal peptide prediction; (**H**) Secondary structure prediction of EBD protein; (**I**) Tertiary structure modeling of EBD protein; (**J**) Phylogenetic analysis of EBD within the AMPs family.

The EBD coding sequence (CDS) was inserted into the eukaryotic expression vector PCDH via homologous recombination, yielding the recombinant plasmid PCDH-EBD with a total length of 8381 bp (Figure 1A). The construct was transformed into competent *Escherichia coli* DH5α cells, and plasmid DNA was extracted. Colony PCR screening of ten independent clones identified four positive clones (PE1, PE2, PE4, and PE10), each producing an amplicon of approximately 194 bp, consistent with the expected fragment size, thus confirming successful insertion of the target gene (Figure 1B).

Genomic localization analysis showed that EBD is situated within an *AMP* gene cluster in the bovine genome, adjacent to defensin family-related genes including *CATHL5*, *CATHL6*, *DEFB1*, and *TAP*, with *LOC507527* and *LOC789175* identified as the nearest neighboring loci (Figure 1C). Physicochemical property analysis indicated that the N-terminal region showed a maximum hydrophobicity score of 3.233, whereas the central and C-terminal regions were predominantly hydrophilic, reaching a minimum value of −0.922; overall, full-length EBD containing the signal peptide was characterized as a hydrophobic protein (Figure 1D). Transmembrane topology prediction using TMHMM 2.0 confirmed the absence of transmembrane helices, classifying EBD as a non-transmembrane protein (Figure 1E).

Post-translational modification analysis using NetPhos 3.1 identified multiple putative serine and threonine phosphorylation sites, suggesting potential regulation via phosphorylation (Figure 1F). Signal peptide prediction using SignalP 4.1 revealed a canonical N-terminal signal peptide, indicating that EBD is a secreted protein (Figure 1G). Secondary structure prediction demonstrated that EBD is composed primarily of α-helices, β-sheets, and random coils (Figure 1H), while tertiary structure modeling confirmed the characteristic structural fold typical of β-defensins (Figure 1I). Phylogenetic analysis based exclusively on homologous defensin sequences placed EBD within the bovine β-defensin clade and showed its close evolutionary relationship with other ruminant β-defensin family members. These results support the classification of EBD as a member of the bovine β-defensin family (Figure 1J).

### 3.2. Cell Transfection of Recombinant Plasmid PCDH-EBD and SDS-PAGE Analysis

**Figure 2:**
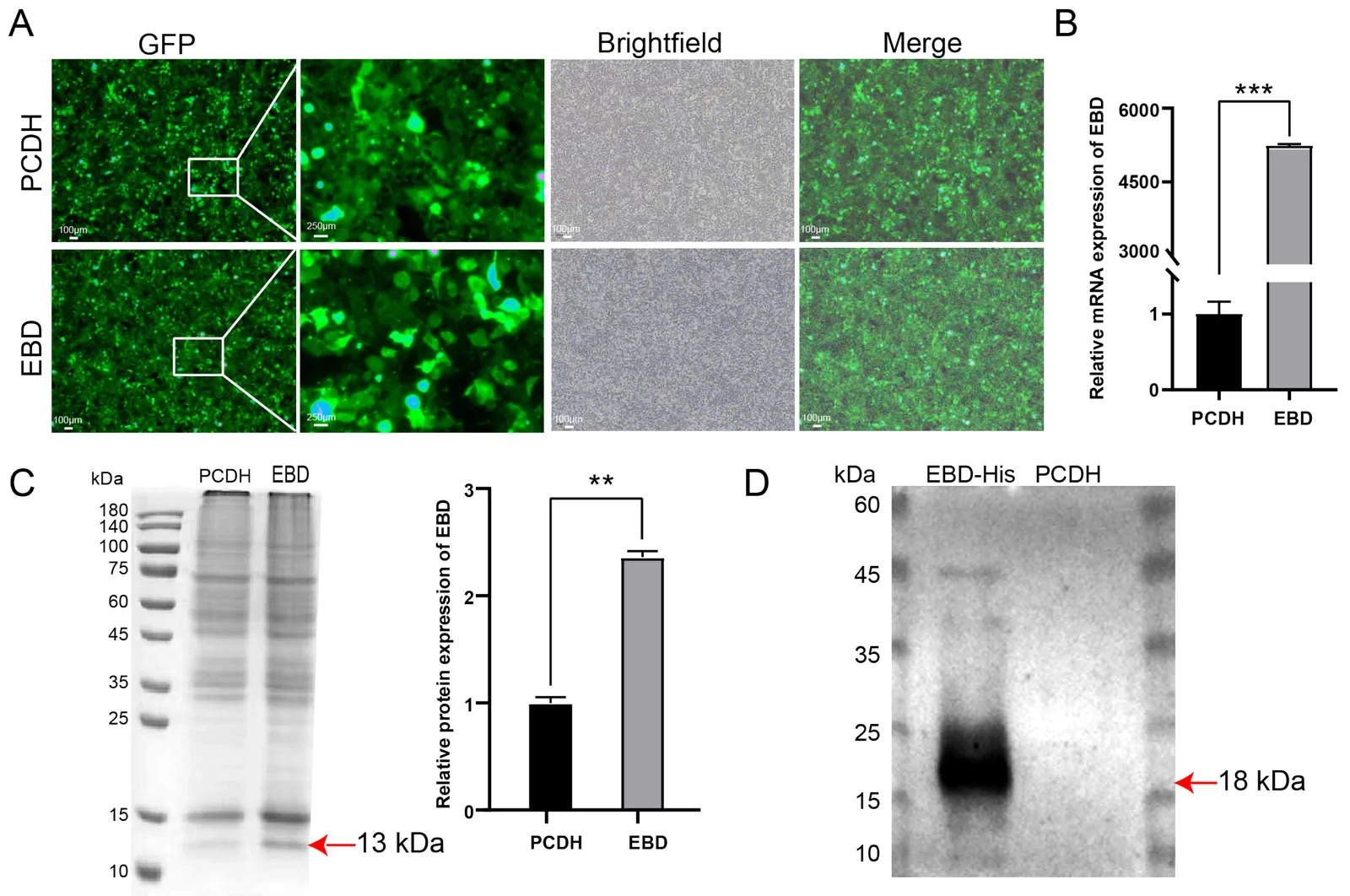
Gene and protein expression analysis of EBD in 293T cells transfected with PCDH-EBD. (**A**) Representative fluorescence microscopy images showing GFP fluorescence, bright-field images, and merged images of 293T cells transfected with the empty PCDH vector or recombinant PCDH-EBD plasmid. (**B**) Relative EBD mRNA expression determined by qRT-PCR. Data are from three independent transfections, each with three technical replicates. (**C**) SDS-PAGE followed by Coomassie Brilliant Blue staining of total cellular protein samples from the PCDH and PCDH-EBD groups and corresponding densitometric quantification. Data are from three independent experiments. (**D**) Western blot analysis of purified EBD-His-tag recombinant protein. Representative blot from three independent experiments is shown. ** *p* < 0.01, *** *p* < 0.001.

To evaluate the expression of EBD from the recombinant PCDH-EBD plasmid in 293T cells, fluorescence microscopy, qRT-PCR, SDS-PAGE, and Western blotting were performed. Fluorescence microscopy showed comparable GFP fluorescence in cells transfected with the empty PCDH vector and those transfected with PCDH-EBD, indicating successful transfection in both groups (Figure 2A). qRT-PCR analysis revealed that EBD mRNA expression was significantly increased in the PCDH-EBD group compared with the empty-vector PCDH group (*p* < 0.001) (Figure 2B). SDS-PAGE followed by Coomassie Brilliant Blue staining showed an enhanced protein band at approximately 13 kDa in the PCDH-EBD group, and densitometric analysis further demonstrated significantly higher expression of the corresponding protein in the PCDH-EBD group than in the PCDH group (*p* < 0.01) (Figure 2C). The total cellular protein samples used for Coomassie-stained SDS-PAGE were treated to remove the His-tag sequence. Western blotting further detected a prominent immunoreactive band at approximately 18 kDa using purified recombinant EBD-His-tag protein from the PCDH-EBD group, whereas no corresponding band was observed in the empty-vector PCDH group (Figure 2D). The discrepancy in apparent molecular weight (13 kDa versus 18 kDa) arises from His-tag removal in total cell lysates for SDS-PAGE, while Western blot was performed on purified protein retaining the His-tag. Collectively, these results demonstrated successful expression of recombinant EBD in 293T cells transfected with PCDH-EBD.

### 3.3. In Vitro Antibacterial Activity and In Vivo Protective Effects of EBD

**Figure 3:**
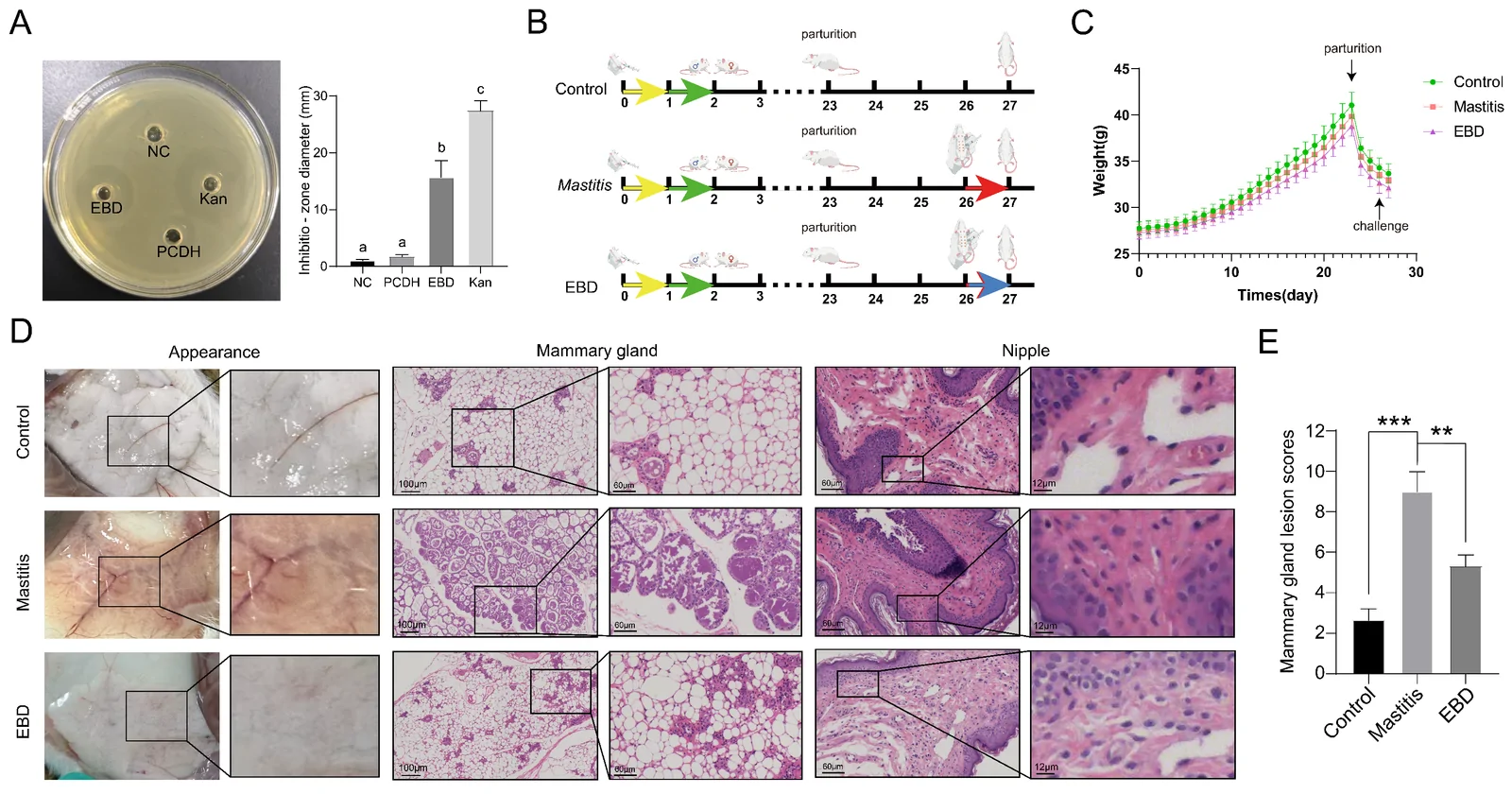
In Vitro Antibacterial Activity and In Vivo Protective Effects of EBD. (**A**) Antibacterial activity of EBD against *E. faecalis* (NC: blank control; P: PCDH negative control; K+: kanamycin positive control; EBD: EBD protein experimental group; Different lowercase letters in panel A indicate statistically significant differences among groups (*p* < 0.05), whereas the same letter indicates no significant difference.); (**B**) Schematic workflow of the animal experiment, In panel (**B**), the yellow arrows indicate pregnant mare serum gonadotropin treatment, the green arrows indicate chorionic gonadotropin treatment, the red arrows indicate *Enterococcus faecalis* treatment, and the blue arrows indicate *Enterococcus faecalis* treatment and EBD protein treatment.; (**C**) Body weight monitoring of mice; (**D**) Gross morphology of mammary glands, H&E staining of mammary gland tissue and H&E staining of teat tissue; (**E**) Mammary gland lesion scores. The total lesion score (range 0–12) was calculated by summing four individual parameters: inflammatory cell infiltration, acinar destruction, interstitial edema, and tissue necrosis, each scored 0–3. *n* = 5 mice per group. Data are mean ± SD. ** *p* < 0.01, *** *p* < 0.001.

The antibacterial activity of recombinant EBD against six bacterial strains was evaluated using the agar well diffusion assay (Figure 3A, Supplementary Figure S1). Inhibition-zone diameters were determined from 5 independent experiments and are presented as mean ± SD in Table 2. EBD protein exhibited varying degrees of growth-inhibitory activity against all tested bacterial strains, with inhibition zone diameters ranging from 9.52 ± 0.18 to 15.78 ± 0.22 mm. The largest inhibition zone was observed against *Enterococcus faecalis* (15.78 ± 0.22 mm), followed by *Escherichia coli* ATCC 25922 (15.14 ± 0.13 mm) and *Staphylococcus aureus* ATCC 29213 (12.28 ± 0.29 mm), indicating moderate antibacterial activity. EBD protein also showed inhibitory activity against *Salmonella enterica* serovar Typhimurium ATCC 14028 (11.67 ± 0.34 mm), *Salmonella enterica* serovar Enteritidis ATCC 13076 (10.33 ± 0.26 mm), and MRSA ATCC 43300 (9.52 ± 0.18 mm), which were classified as weak antibacterial activity according to the predefined inhibition zone criteria. Compared with the kanamycin positive control, the EBD-containing preparation produced a smaller inhibition zone against *E. faecalis*, indicating lower apparent growth-inhibitory activity under the tested conditions. These results indicate that the EBD-containing preparation exhibited growth-inhibitory activity against the tested Gram-positive and Gram-negative bacterial strains under the agar diffusion assay conditions.

Following the evaluation of the in vitro antibacterial activity of recombinant EBD, its protective effects were further investigated in an *E. faecalis*-induced murine mastitis model. The experimental workflow is presented in Figure 3B, in which a pregnant mouse model was established, and interventions were administered after parturition. Body weight measurements indicated normal gestational weight gain followed by physiological postpartum weight loss across all groups, with no significant differences among groups (Figure 3C). Histological examination of H&E-stained mammary sections showed that control glands had intact acinar architecture, a single-layered epithelium, and few inflammatory cells, corresponding to lesion score 0. In contrast, mastitic glands exhibited predominant neutrophil infiltration, accompanied by macrophages and scattered lymphocytes, together with acinar destruction, interstitial edema, and focal necrosis, corresponding to lesion scores 8–9. EBD-treated glands showed reduced neutrophil and macrophage infiltration and partial restoration of acinar architecture, corresponding to lesion scores 4–5.

Mammary lesion scoring demonstrated that the model group had significantly higher lesion scores than the control group (*p* < 0.001), while EBD treatment significantly reduced these scores (*p* < 0.01) (Figure 3E).

### 3.4. Effects of Bovine Enteric β-Defensin on the Mammary Tissue-Associated Microbiota in Mice with Mastitis

**Figure 4:**
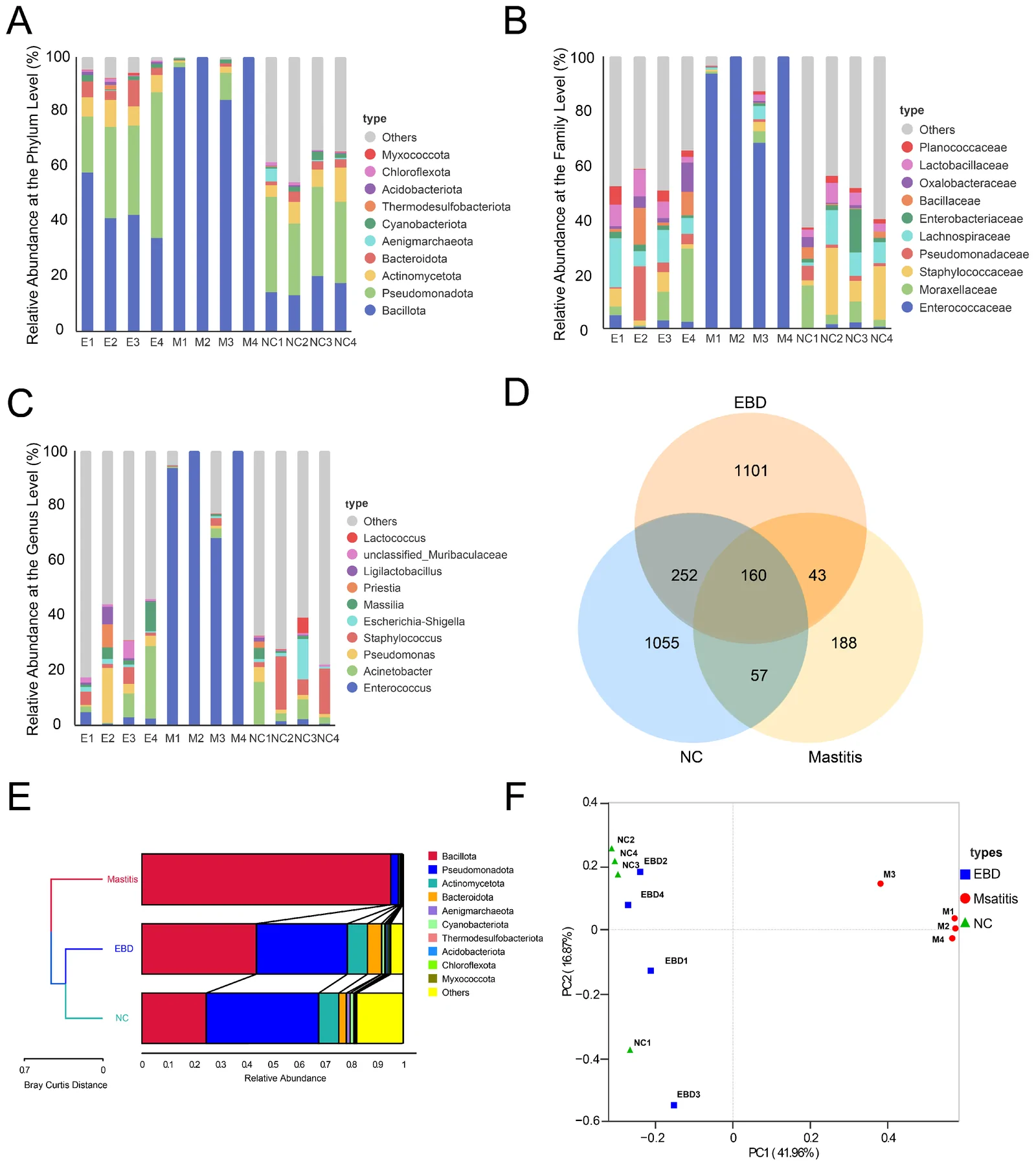
Taxonomic composition and community structure of mammary tissue-associated microbiota in mice with *E. faecalis*-induced mastitis. (**A**–**C**) Relative taxonomic composition of bacterial communities at the phylum (**A**), family (**B**), and genus (**C**) levels. Each stacked bar represents an individual mammary tissue sample, and different colors indicate different bacterial taxa. Samples were obtained from the NC, Mastitis, and EBD-treated groups (*n* = 4 per group). (**D**) Venn diagram illustrating shared and unique amplicon sequence variants (ASVs) among the three groups. (**E**) UPGMA clustering based on microbial community composition. (**F**) Principal coordinate analysis (PCoA) illustrating differences in bacterial community structure among samples.

To characterize the mammary tissue-associated bacterial communities, 16S rRNA gene sequencing was performed on mammary gland tissue samples collected from the NC, Mastitis, and EBD-treated groups (*n* = 4 per group). The relative taxonomic composition of the bacterial communities was examined at the phylum, family, and genus levels (Figure 4A–C). The stacked bar plots display the relative abundances of bacterial taxa in individual samples, allowing visualization of both within-group variability and differences in microbial composition among the experimental groups.

At the phylum level, Firmicutes and Proteobacteria were among the dominant bacterial phyla detected in the mammary tissue samples (Figure 4A). Family- and genus-level analyses further illustrated differences in the distribution of bacterial taxa among samples (Figure 4B,C). In particular, the relative abundance patterns of *Enterococcus* appeared to differ between the Mastitis and EBD-treated groups. These descriptive observations were subsequently evaluated using differential abundance analysis to determine whether the observed differences were statistically supported.

The Venn diagram illustrated both group-specific and shared amplicon sequence variants (ASVs) among the NC, Mastitis, and EBD-treated groups (Figure 4D), indicating differences in the detected ASV composition across the three experimental conditions. UPGMA clustering (Figure 4E) and PCoA (Figure 4F) were used to visualize differences in mammary tissue-associated microbial community structure among groups. Pairwise Adonis analysis based on Bray–Curtis distances indicated significant differences between the EBD and Mastitis groups (pseudo-F = 6.5875, *R*^2^ = 0.5233, *p* = 0.030) and between the Mastitis and NC groups (pseudo-F = 9.4797, *R*^2^ = 0.6124, *p* = 0.030), whereas no significant difference was detected between the EBD and NC groups (pseudo-F = 1.2862, *R*^2^ = 0.1765, *p* = 0.158).

**Figure 5:**
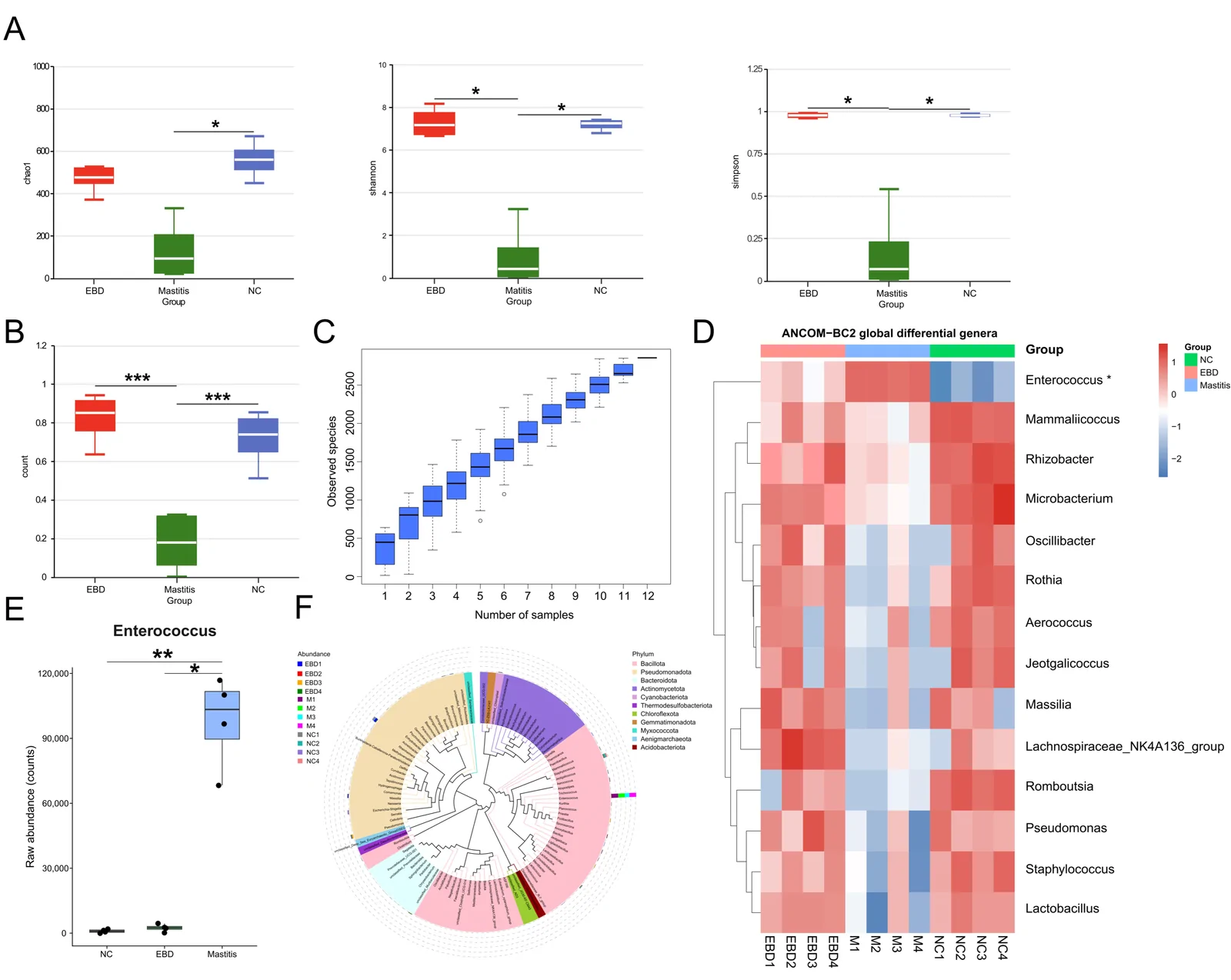
EBD treatment modulates the mammary gland microbiota in mice with *Enterococcus faecalis*-induced mastitis. (**A**) Alpha diversity analysis, including Chao1, Shannon, and Simpson indices; (**B**) Beta diversity analysis showing inter-group differences; (**C**) Species accumulation curve analysis; (**D**) Heatmap of genera identified by ANCOM-BC2 global analysis; asterisks denote genera with FDR-adjusted *q* < 0.05 from ANCOM-BC2 analysis. (**E**) Abundance of *Enterococcus* among the three groups. (**F**) Genus-level phylogenetic tree. ANCOM-BC2 significance was assessed using FDR-adjusted *q* values. * *p* < 0.05, ** *p* < 0.01, and *** *p* < 0.001.

Alpha diversity analysis showed a reduction in the Shannon index in the Mastitis group compared with the NC group, whereas the infected EBD-treated group exhibited a higher Shannon index than the untreated Mastitis group (Figure 5A). These findings indicate that EBD administration was associated with differences in mammary tissue-associated microbial diversity under *E. faecalis* challenge. However, because an uninfected EBD-only group was not included, the independent effects of EBD on microbial diversity under physiological conditions could not be determined. To further determine whether mastitis-associated microbial alterations were reversed by EBD treatment, differential abundance analysis was performed using ANCOM-BC2, focusing on three biologically relevant pairwise comparisons: Mastitis versus NC, EBD versus Mastitis, and EBD versus NC. The global ANCOM-BC2 analysis identified 14 genera with significant differences among the three groups (Figure 5D). In particular, *Enterococcus* showed marked enrichment in the Mastitis group compared with NC (LFC = 5.97, FDR-adjusted *q* = 0.0062) and was reduced in the EBD group relative to the Mastitis group (Mastitis vs. EBD: LFC = 5.48, *q* = 0.0163), whereas EBD and NC showed no significant difference (EBD vs. NC: LFC = 0.49, *q* = 1.000). These results indicate that EBD treatment was associated with a lower relative abundance of *Enterococcus* compared with the Mastitis group (Figure 5E).

Pairwise differences in community composition were observed for selected group comparisons; however, these findings should be interpreted cautiously because of the small sample size (Figure 5B). Species accumulation curves approached saturation, indicating that the sequencing effort was sufficient to capture most of the microbial diversity represented in the samples (Figure 5C), and genus-level phylogenetic analysis further illustrated the differential distribution of dominant taxa across treatment conditions (Figure 5F). Collectively, the pairwise ANCOM-BC2 comparisons addressed the treatment-related microbial trajectory from NC to Mastitis and subsequently to EBD treatment: mastitis was associated with pronounced alterations in specific bacterial taxa, while EBD treatment shifted these alterations toward the NC profile. The particularly strong enrichment of *Enterococcus* in the Mastitis group and its reduction following EBD treatment were consistent with the *E. faecalis*-induced mastitis model. These findings suggest that EBD treatment was associated with changes in the mammary tissue-associated microbial community toward the NC group profile.

### 3.5. Mammary Transcriptomic Analysis and qPCR Validation of E. faecalis-Induced Murine Mastitis Following EBD Treatment

**Figure 6:**
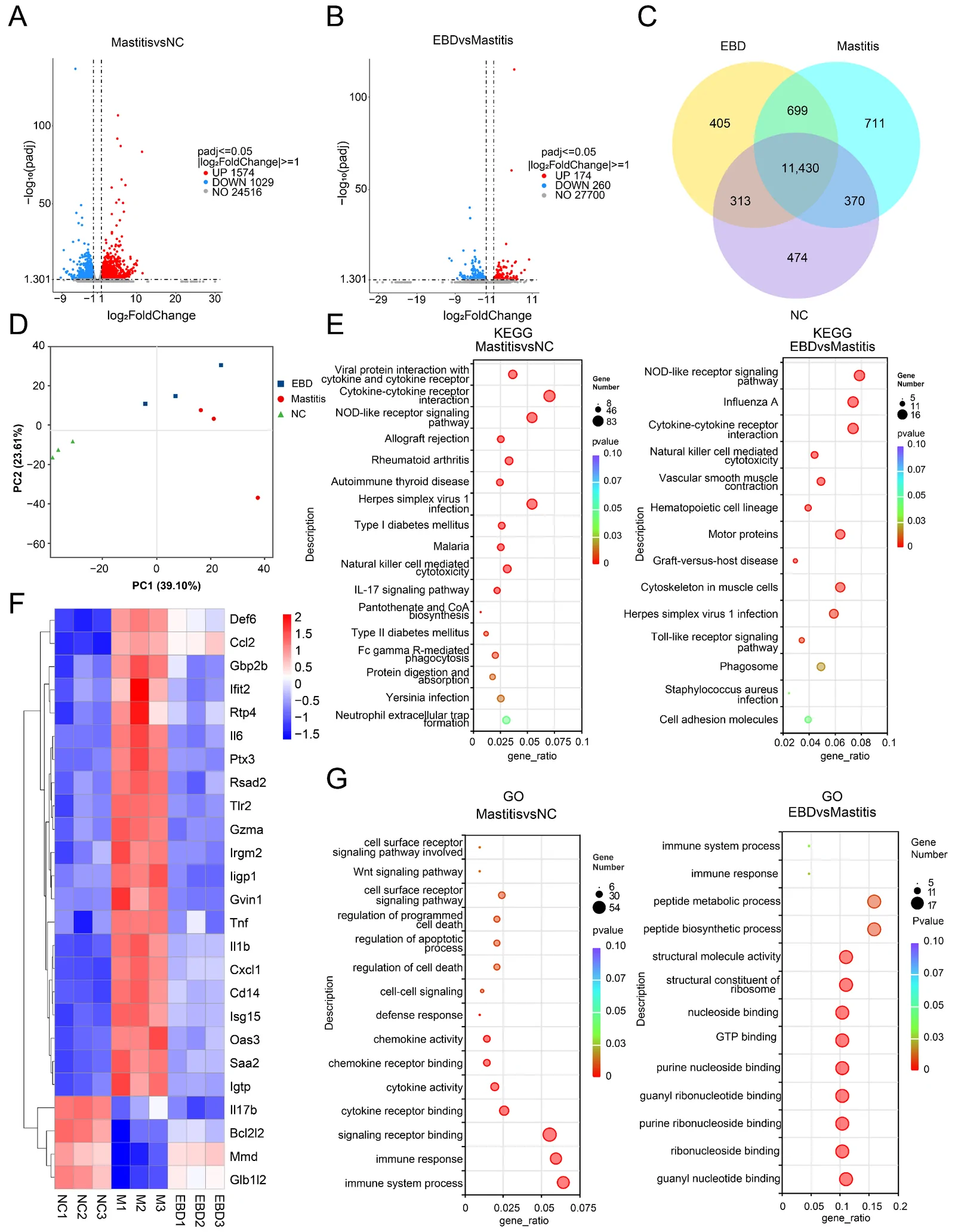
Mammary transcriptomic profiling of recombinant PCDH-EBD in *E. faecalis*-induced murine mastitis. (**A**) Volcano plot of DEGs in the Mastitis vs. NC comparison; (**B**) Volcano plot of DEGs in the EBD vs. Mastitis comparison; (**C**) Venn diagram showing shared and unique expressed genes among groups; (**D**) Principal component analysis (PCA) of transcriptomic profiles; (**E**) KEGG pathway enrichment analysis of DEGs; (**F**) Hierarchical clustering heatmap of DEGs; (**G**) GO enrichment analysis of DEGs.

RNA-seq was performed on mammary gland tissues collected from the NC, Mastitis, and EBD groups to investigate the transcriptional responses associated with EBD treatment. Differential expression analysis revealed significant transcriptomic alterations in mastitic mammary tissue. In the Mastitis versus NC comparison, 1574 genes were significantly upregulated and 1029 genes were significantly downregulated according to the criteria of |log_2_FoldChange| ≥ 1 and Benjamini–Hochberg-adjusted *p*-value ≤ 0.05 (Figure 6A). In the EBD versus Mastitis comparison, 174 genes were upregulated and 260 genes were downregulated (Figure 6B), demonstrating a distinct transcriptional response associated with EBD administration. However, 1509 upregulated and 1254 downregulated genes were also identified in the EBD versus NC comparison (Supplementary Figure S2), indicating that the overall transcriptional profile of EBD-treated mammary tissue remained different from that of uninfected controls. Therefore, the data support changes in selected inflammatory and host-response pathways rather than global normalization of the mammary transcriptome. Venn diagram analysis identified 11,430 genes commonly expressed among all groups, whereas 405, 711, and 474 genes were uniquely expressed in the EBD, Mastitis, and NC groups, respectively, highlighting distinct transcriptional characteristics among treatment conditions (Figure 6C). PCA demonstrated clear separation of the three groups. Samples from the Mastitis group were separated from those of the NC group, whereas EBD-treated samples showed a shift toward the NC cluster in the PCA plot (Figure 6D). KEGG pathway enrichment analysis of the Mastitis versus NC comparison showed significant enrichment of several inflammation- and immune-related pathways, including the NOD-like receptor (NLR), TNF signaling pathway, and NF-κB signaling pathways. In the EBD versus Mastitis comparison, differentially expressed genes were also enriched in NLR signaling- and phagosome-related pathways, together with pathways involving cytokine–cytokine receptor interactions, regulation of the actin cytoskeleton, and cell adhesion molecules (Figure 6E). These transcriptomic changes suggest that NLR signaling and phagosome-related processes may be involved in the response to EBD treatment. However, because pathway enrichment analysis reflects associations at the transcriptomic level rather than direct pathway activation, these findings should be considered hypothesis-generating and require further experimental validation at the cellular and protein levels. Hierarchical clustering of DEGs demonstrated significant up-regulation of pro-inflammatory genes, including Il6, Il1b, Tnf, and Cxcl1, in the Mastitis group, confirming successful establishment of the inflammatory model (Figure 6F). EBD treatment significantly reduced the expression of these inflammatory mediators. *n* = 3 mice per group. GO enrichment analysis identified differences in biological processes and molecular functions associated with the differentially expressed genes between the EBD and Mastitis groups. KEGG enrichment analysis further identified NOD-like receptor signaling and phagosome-related pathways among the enriched categories. These findings indicate transcriptomic associations with these pathways but do not establish their activation, inhibition, or causal involvement in the response to EBD treatment. Compared with the Mastitis group, the EBD group displayed enrichment patterns associated with peptide metabolic processes, structural molecular activity, and nucleotide binding, indicating differences in the functional categories represented by the differentially expressed genes (Figure 6G).

**Figure 7:**
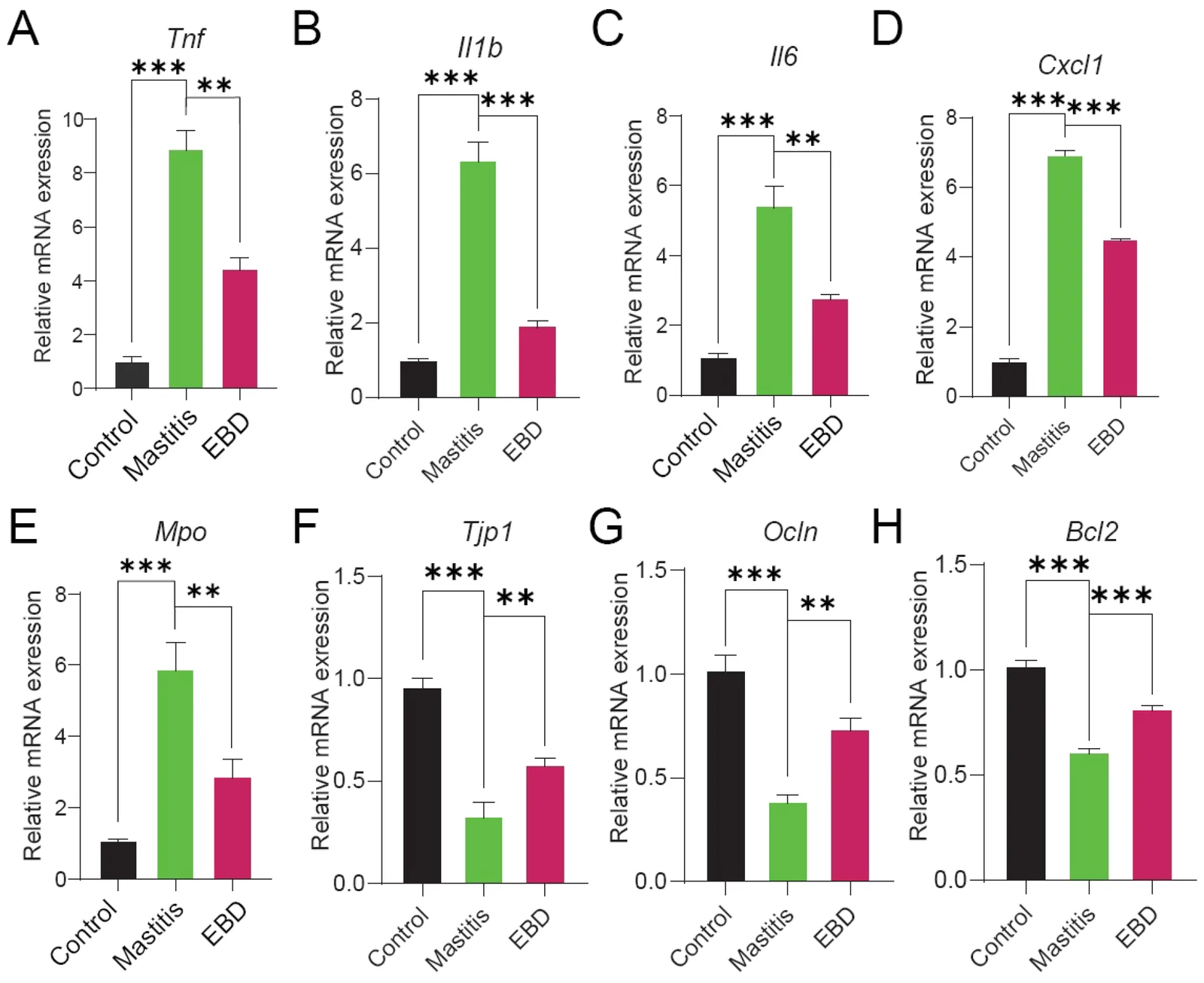
RT-qPCR validation of representative differentially expressed genes identified by transcriptomic analysis. Relative mRNA expression levels of (**A**) *Tnf*, (**B**) *Il1b*, (**C**) *Il6*, (**D**) *Cxcl1*, (**E**) *Mpo*, (**F**) *Tjp1*, (**G**) *Ocln*, and (**H**) *Bcl2* in mammary tissues from the Control, Mastitis, and EBD-treated groups. Gene expression was normalized to *Actb*. Data are presented as mean ± SD. ** *p* < 0.01, *** *p* < 0.001.

To validate the reliability of the transcriptomic analysis, representative differentially expressed genes associated with inflammation, neutrophil recruitment, epithelial barrier integrity, and apoptosis were selected for RT-qPCR analysis. As shown in Figure 7, the mRNA expression levels of the pro-inflammatory and neutrophil-associated genes *Tnf*, *Il1b*, *Il6*, *Cxcl1*, and *Mpo* were markedly increased in the Mastitis group compared with the Control group, whereas EBD treatment significantly reduced their expression. In contrast, the expression levels of the epithelial barrier-related genes *Tjp1* and *Ocln*, as well as the anti-apoptotic gene *Bcl2*, were decreased in the Mastitis group and partially restored following EBD treatment. Overall, the RT-qPCR results were consistent with the expression trends observed in the transcriptomic analysis, supporting the reliability of the sequencing data and further indicating that EBD treatment attenuated the inflammatory response while promoting recovery of mammary epithelial barrier-associated and cell survival-related gene expression.

## 4. Discussion

**Figure 8:**
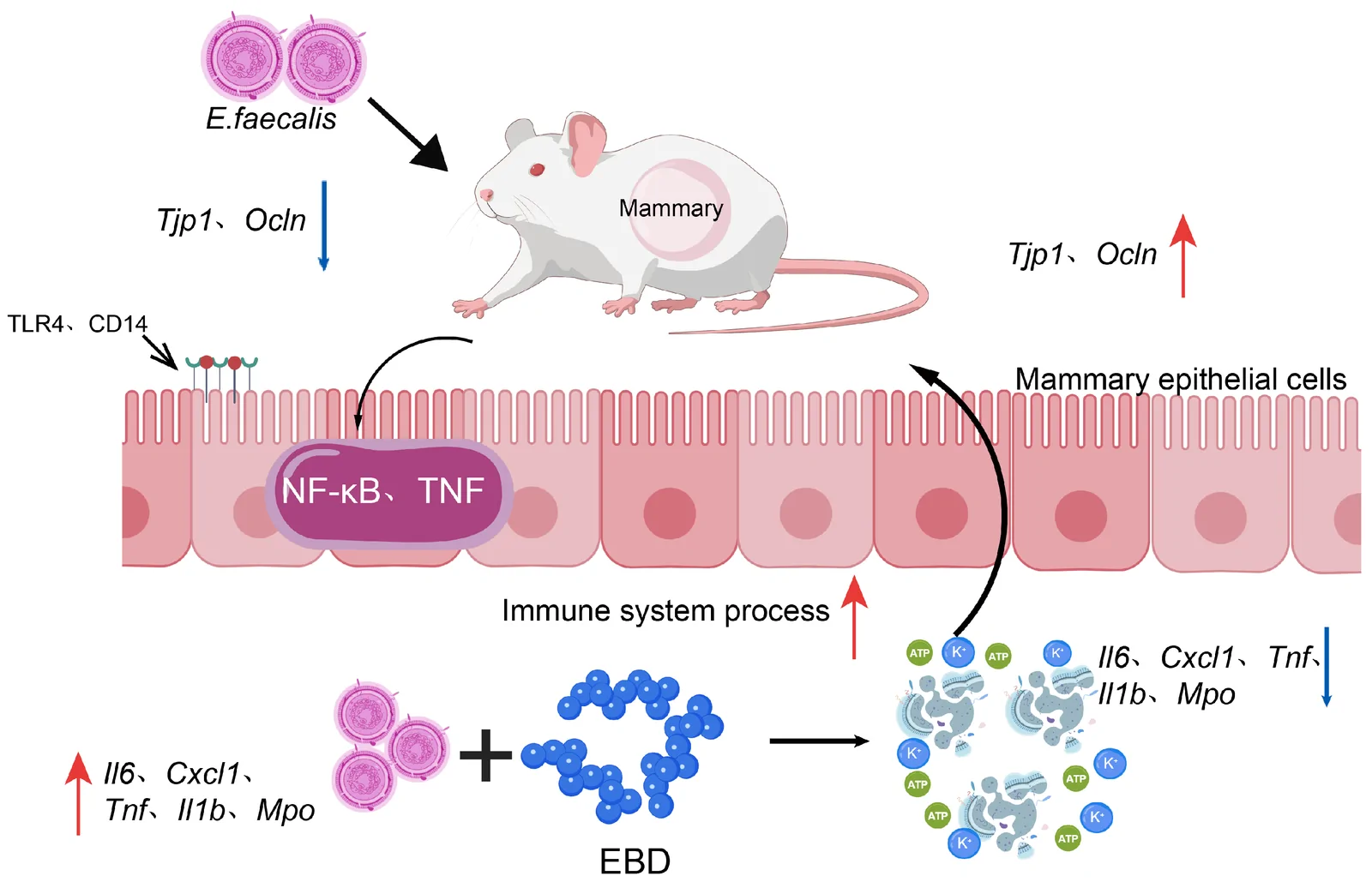
Proposed hypothetical model of the protective effects of bovine β-defensin EBD in *E. faecalis*-induced murine mastitis.

Bovine β-defensins are important components of innate immunity and contribute to antimicrobial defense and immune regulation in the mammary gland [17]. Previous studies have highlighted the potential of host-defense peptides as alternatives or adjuncts to conventional antimicrobial strategies for bovine mastitis [18,19,20]. However, the antibacterial activity and protective effects of Xinjiang Brown Cattle-derived enteric β-defensin (EBD) against *Enterococcus faecalis*-associated mammary inflammation remain poorly characterized. In this study, we evaluated the antibacterial activity of a recombinant EBD-containing preparation and its effects in a murine model of mammary inflammation. The preparation inhibited bacterial growth in vitro, while its co-administration with *E. faecalis* was associated with reduced mammary tissue injury and inflammatory gene expression. Microbiome and transcriptomic analyses further identified accompanying changes in mammary tissue-associated bacterial composition and host-response pathways. A schematic summary of the observed antibacterial and protective effects associated with EBD administration is presented in Figure 8. Together, these findings provide preliminary evidence supporting further investigation of bovine EBD as a host-defense peptide candidate.

The antibacterial findings can be considered alongside previous studies of alternative antimicrobial approaches for mastitis control. *E. faecalis* is an opportunistic mastitis-associated pathogen whose antimicrobial resistance and virulence characteristics can complicate infection management [21]. Field et al. [10] reported enhanced anti-staphylococcal activity of bioengineered nisin variants, whereas Ji et al. [16] demonstrated that bacteriophage EF-N13 reduced bacterial burden and mammary inflammation in a murine model challenged with multidrug-resistant *E. faecalis*. Antimicrobial peptides have also shown activity against resistant enterococci, supporting their further investigation as antibacterial candidates [22]. Recent studies have further highlighted the antibacterial potential of host-defense peptides. Cho et al. (2024) demonstrated that several antimicrobial peptides exhibited broad-spectrum activity against bovine mastitis-associated pathogens, including *E. faecalis* [23]. Furthermore, Saubi et al. (2025) reported that recombinant bovine β-defensins exhibited distinct antimicrobial and immunomodulatory properties, with BNBD4 showing notable antibacterial activity against *E. coli* [24]. These findings support further investigation of bovine β-defensins as potential antimicrobial candidates, although their activities may differ according to peptide identity and target bacterial species. In contrast to the pathogen specificity of bacteriophages, the EBD-containing preparation exhibited growth-inhibitory activity against several tested Gram-positive and Gram-negative bacterial strains. However, inhibition-zone diameters varied among strains, and the zone observed against *E. faecalis* was smaller than that produced by the kanamycin control. Because agar diffusion results are influenced by assay conditions and molecular diffusion, these findings do not establish the comparative antibacterial potency of EBD. Standardized minimum inhibitory concentration, minimum bactericidal concentration, and time-kill assays are needed to characterize its activity more precisely.

In the murine model, administration of the EBD-containing preparation was associated with reduced histopathological injury and lower expression of inflammatory mediators, including *Tnf*, *Il1b*, *Il6*, *Cxcl1*, and *Mpo*. These observations are consistent with the broader roles of bovine β-defensins and other host-defense peptides in antimicrobial defense and immune regulation [17,18]. Changes in the expression of *Tjp1*, *Ocln*, and *Bcl2* also suggested potential involvement of epithelial barrier- and cell survival-related processes. Excessive inflammatory signaling, including NF-κB-associated responses, is recognized as an important contributor to mammary inflammation [25]. In addition, previous studies using other anti-inflammatory interventions have demonstrated that modulation of inflammatory responses can attenuate mammary tissue injury in experimental models [26]. Nevertheless, the present results do not establish direct regulation of these signaling mechanisms by EBD, and changes in mRNA expression alone cannot demonstrate restoration of epithelial barrier function or inhibition of apoptosis. Furthermore, because EBD and the bacterial inoculum were administered simultaneously, the observed effects should be interpreted as early protection under experimental challenge conditions rather than therapeutic efficacy against established mastitis.

The microbiome and transcriptomic findings provided complementary information about the biological responses associated with EBD administration. Mammary tissue-associated 16S rRNA sequencing showed an increased relative abundance of *Enterococcus* in the Mastitis group and a lower relative abundance in the EBD-treated group. However, relative abundance data do not establish the absolute number or viability of the inoculated bacteria. These findings also require cautious interpretation because mammary tissue is a relatively low-biomass sampling environment, and the number of biological replicates was limited. Transcriptomic analysis identified treatment-associated changes involving immune-response and phagosome-related pathways, including NOD-like receptor signaling. Such observations are compatible with the known involvement of innate immune processes in host defense [27], but pathway enrichment does not demonstrate direct pathway activation, inhibition, or causal regulation by EBD. Importantly, the EBD-treated group retained substantial transcriptional differences from uninfected controls, indicating that the findings support changes in selected host-response processes rather than global normalization of the mammary transcriptome.

Several limitations should be considered. The antibacterial assay was conducted at a single preparation concentration, without standardized potency or dose–response measurements. Further characterization of recombinant EBD identity, purity, and specific activity is also required. The animal experiment involved a small sample size, a single 24 h endpoint, and simultaneous administration of EBD and bacteria. Moreover, an infected group receiving a formulation-matched preparation obtained from empty-vector-transfected cells was not included, limiting the ability to attribute the observed effects exclusively to EBD. The microbiome and transcriptomic analyses were exploratory, and neither absolute bacterial quantification nor targeted mechanistic experiments were performed. These limitations restrict conclusions about bacterial clearance, direct immunomodulation, and therapeutic efficacy.

From a practical perspective, the present findings identify a bovine-derived peptide candidate worthy of further investigation within the broader development of alternatives to conventional mastitis treatments [28]. If its antibacterial activity and safety can be confirmed, purified EBD may be considered for evaluation in locally administered or adjunctive formulations. Recent research has also explored formulation-based approaches to enhance the efficacy of bovine-derived antimicrobial peptides. Wang et al. (2026) demonstrated that chitosan nanoparticle delivery improved the antibacterial activity of bovine lactoferricin and alleviated mammary inflammation in a murine mastitis model [29]. Although these findings cannot be directly extrapolated to EBD, they highlight the potential importance of peptide stability and delivery strategies in the development of antimicrobial peptide-based interventions for mastitis. However, future studies must establish its potency against additional clinical isolates, determine whether delayed administration is effective after infection onset, and assess peptide stability and activity in milk. Studies of cytotoxicity, local mammary tissue tolerance, formulation suitability, and food-safety implications will also be necessary. Ultimately, controlled experiments in lactating dairy cattle are required to determine whether the preliminary protective responses observed in mice can be translated into an effective mastitis intervention.

## 5. Conclusions

This study cloned and expressed Xinjiang Brown Cattle-derived enteric β-defensin (EBD) and demonstrated growth-inhibitory activity of an EBD-containing preparation against several bacterial strains in vitro. In a murine model of *Enterococcus faecalis*-induced mammary inflammation, co-administration of the preparation was associated with reduced histopathological injury and lower expression of inflammation-related genes. Microbiome and transcriptomic analyses identified accompanying changes in mammary tissue-associated bacterial composition and host-response pathways. These findings support further investigation of EBD as a potential candidate for mastitis control. However, its specific antibacterial potency, efficacy against established infection, underlying mechanisms, and safety and effectiveness in dairy cattle remain to be determined.

## Figures and Tables

**Table 1:** Primer sequences used for RT-qPCR analysis.

Target	Forward Primer	Reverse Primer
*Actb*	AGGATTCCTATGTGGGCGA C	ATAGCACAGCCTGGATAGCAA
EBD	CTGGTACTGTCTGCTGGGT C	GCGTGAAACAGGTGCCAATC
*Mpo*	GCCCGCATTCCTTGTTTTCT GGCAG	TTCTCCCCATTCCATCGAGGATTCAGG
*Tnf*	GCGCTGAGGTCAATCTGCCCAAGTACTTA	GCTGGGTAGAGAATGGATGAACA CCCA
*Il6*	TGTATGAACAACGATGATG CA	TCCAGAAGACCAGAGGAAATTTT
*Cxcl1*	AACCGAAGTCATAGCCAC A	GGGGACACCTTTTAGCATC
*Il1b*	TGTCCTGATGAGAGCAT CC	AAGGTCCACGGGAAAGAC
*Tjp1*	ACCATGCCTAAAGCTGTCC	GGAACTCAACACACCACCA
*Ocln*	CCTACTCCTCCAATGGCAAA	CTCTTGCCCTTTCCTGCTTT
*Bcl2*	ACTTCTCTCGTCGCTACCGT	TGGGGCCATATAGTTCCACAA

**Table 2:** Antibacterial activity of EBD protein against different bacterial strains.

Strains	Bacterial Species	Inhibition Zone Diameter (mm)	Antibacterial Activity
ATCC 25922	*Escherichia coli*	15.14 ± 0.13	++
ATCC 14028	*Salmonella enterica* serovar Typhimurium	11.67 ± 0.34	+
ATCC 29213	*Staphylococcus aureus*	12.28 ± 0.29	++
ATCC 43300	MRSA	9.52 ± 0.18	+
ATCC 13076	*Salmonella enterica* serovar Enteritidis	10.33 ± 0.26	+
natural cases	*Enterococcus faecalis*	15.78 ± 0.22	++

Data are expressed as mean ± SD from five independent experiments (*n* = 5). Antibacterial activity was classified according to the inhibition zone diameter: <8 mm, negative; 8–12 mm, weak (+); 12–16 mm, moderate (++).

## Data Availability

The raw 16S rRNA gene sequencing reads generated in this study have been deposited in the NCBI Sequence Read Archive under BioProject accession number PRJNA1511050. The raw transcriptome sequencing reads generated in this study have been deposited in the NCBI Sequence Read Archive (SRA) database under BioProject accession number PRJNA1510509. All deposited datasets will be released upon article acceptance.

## Supplementary Materials

The following supporting information can be downloaded at: https://www.mdpi.com/article/10.3390/cells15211980/s1. Figure S1: Representative images of the antibacterial activity of the recombinant EBD-containing preparation against different bacterial strains determined using the agar well diffusion assay; Figure S2: Volcano plot of differentially expressed genes (DEGs) between the EBD-treated and normal control (NC) groups.
