# Supplementary material for: Xinjiang Brown Cattle-Derived β-Defensin EBD Alleviates Enterococcus faecalis-Induced Mammary Inflammation in Mice
Source: Cells. 2026 Oct 30;15(21):1980. doi: 10.3390/cells15211980 (PMC13762411; doi:10.3390/cells15211980)
Supplement: Supplementary file 1 [file cells-15-01980-s001.zip › cells-4581375-supplementary.pdf]

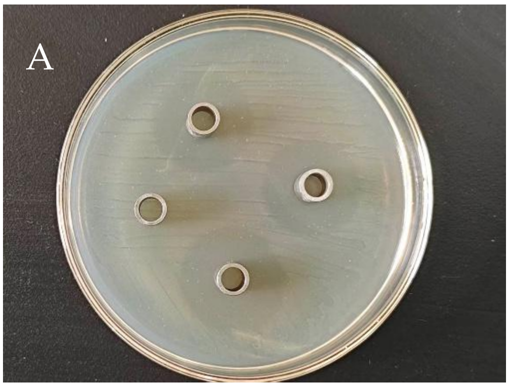

ATCC 13076 Salmonella enterica
serovar Enteritidis

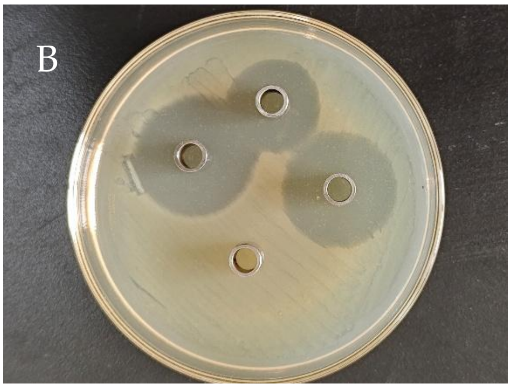

ATCC 14028 Salmonella enterica
serovar Typhimurium

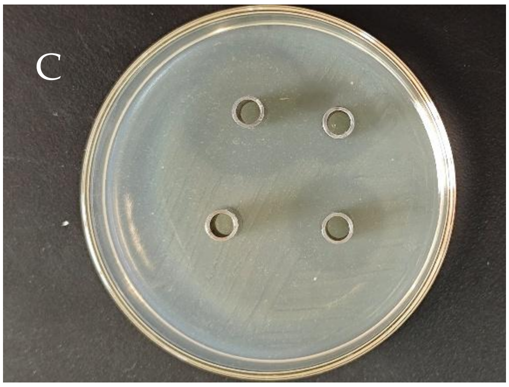

ATCC 25922
Escherichia coli

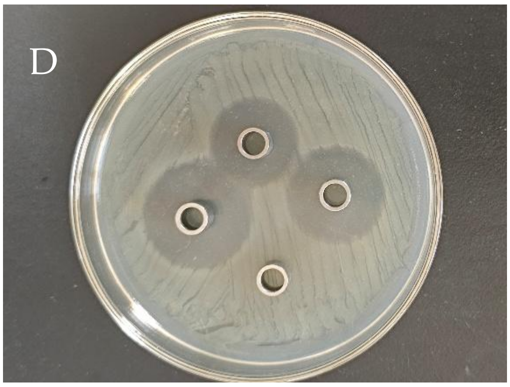

ATCC 29213
Staphylococcus aureus

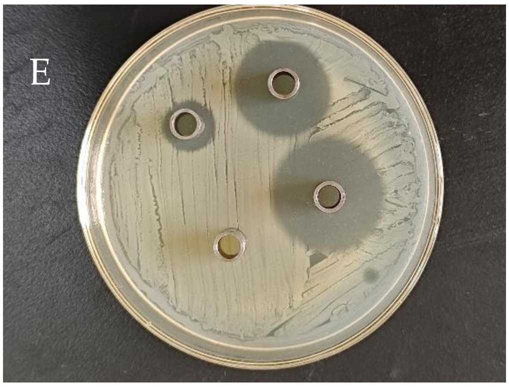

ATCC 43300
MRSA

Figure S1. Representative images of the antibacterial activity of the recombinant EBD-containing preparation against different bacterial strains determined using the agar well diffusion assay. (A) *Salmonella enterica* subsp. *enterica* serovar Enteritidis ATCC 13076; (B) *Salmonella enterica* subsp. *enterica* serovar Typhimurium ATCC 14028; (C) *Escherichia coli* ATCC 25922; (D) *Staphylococcus aureus* ATCC 29213; (E) methicillin-resistant *Staphylococcus aureus* (MRSA) ATCC 43300; The treatments included sterile water (blank control), PCDH-derived protein preparation (negative control), EBD-containing preparation (experimental group), and kanamycin (positive control). Images are representative of five independent experiments.

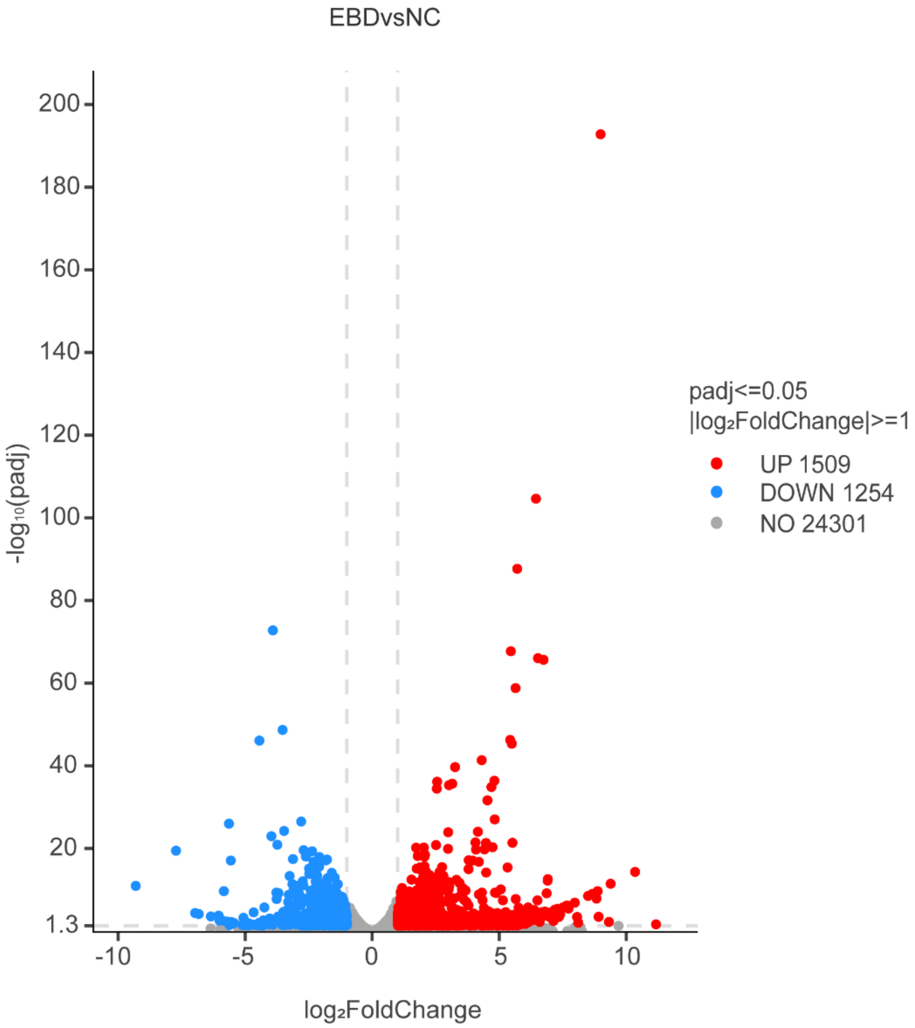

Figure S2. **Volcano plot of differentially expressed genes (DEGs) between the EBD-treated and normal control (NC) groups.**
